# Improved Photoelectrochemical Performance of WO_3_/BiVO_4_ Heterojunction Photoanodes via WO_3_ Nanostructuring

**DOI:** 10.1021/acsami.3c10869

**Published:** 2023-11-03

**Authors:** Chiara Nomellini, Annalisa Polo, Camilo A. Mesa, Ernest Pastor, Gianluigi Marra, Ivan Grigioni, Maria Vittoria Dozzi, Sixto Giménez, Elena Selli

**Affiliations:** † Dipartimento di Chimica, Università degli Studi di Milano, Via C. Golgi 19, I-20133 Milano, Italy; ‡ Institute of Advanced Materials (INAM), Universitat Jaume I, Avenida de Vicent Sos Baynat, S/N, 12006 Castelló, Spain; § IPR−Institut de Physique de Rennes, CNRS, UMR 6251 Université de Rennes, 35000 Rennes, France; ∥ Eni S.p.A Novara Laboratories (NOLAB) Renewable, New Energies and Material Science Research Center (DE-R&D) Via G. Fauser 4, I-28100 Novara, Italy

**Keywords:** tungsten trioxide, bismuth vanadate, heterojunction, photoanodes, water oxidation, nanostructuring

## Abstract

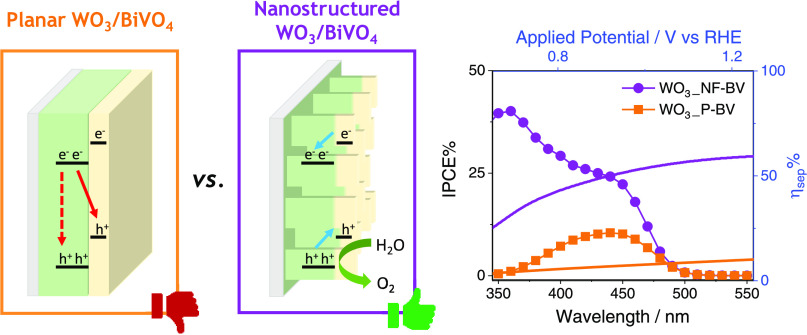

WO_3_/BiVO_4_ heterojunction photoanodes can be efficiently employed in photoelectrochemical (PEC) cells for the conversion of water into molecular oxygen, the kinetic bottleneck of water splitting. Composite WO_3_/BiVO_4_ photoelectrodes possessing a nanoflake-like morphology have been synthesized through a multistep process and their PEC performance was investigated in comparison to that of WO_3_/BiVO_4_ photoelectrodes displaying a planar surface morphology and similar absorption properties and thickness. PEC tests, also in the presence of a sacrificial hole scavenger, electrochemical impedance analysis under simulated solar irradiation, and incident photon to current efficiency measurements highlighted that charge transport and charge recombination issues affecting the performance of the planar composite can be successfully overcome by nanostructuring the WO_3_ underlayer in nanoflake-like WO_3_/BiVO_4_ heterojunction electrodes.

## 1 Introduction

The roadmap toward a sustainable society requires the large-scale deployment of renewable energy technologies, among which hydrogen produced from solar energy is one of the most promising alternatives to fossil fuels, given the abundance of water and the inexhaustibility of solar radiation.^1−3^ Photoelectrochemical (PEC) water splitting with transition-metal oxides has been extensively studied to efficiently convert solar energy and store it in the form of chemical bonds, due to the Earth abundance and stability of many semiconductor oxide photocatalysts under the relatively harsh water oxidation conditions.^4−6^

However, all semiconductor oxides most largely employed as photoanode materials, such as BiVO_4_, WO_3_, and Fe_2_O_3_, suffer from slow water oxidation kinetics and fast recombination of photogenerated electron/hole couples.^4,7−10^ Photocatalyst doping and/or surface modification, combined with heterojunction formation, were found effective strategies to accelerate the rate-determining step of overall water splitting.^11−18^ One outstanding example is the composite material obtained by coupling WO_3_ with BiVO_4_, which combines the excellent visible light harvesting properties of bismuth vanadate with the good electron conductivity of tungsten oxide. Moreover, due to the favorable band alignment in this material, photogenerated charge separation is enhanced, as proved by the increased charge carriers lifetime.^19−21^

To further increase the efficiency of these materials, specific effects derived from nanoscaling can also be exploited. In general, heterojunctions can be either (i) planar, i.e., composed of densely packed aggregates and, potentially, well-oriented facets, or (ii) nanostructured, exhibiting a controlled surface morphology or asperities. Indeed, nanostructuring ensures increased light absorption, due to light scattering effects, and an enhanced contact surface area, favoring charge transfer at the interface.^16,22,23^ Nanostructured WO_3_/BiVO_4_ heterojunctions with various morphologies have been studied in recent years,^24,25^ consisting, for instance, of vertically aligned WO_3_ nanorods or nanowires coated with a thin bismuth vanadate layer,^15,16,26−28^ and exhibiting more or less increased photoactivity compared to planar WO_3_/BiVO_4_ heterojunctions.

Different synthetic routes adopted to fabricate such architectures led to variable performance records for PEC water oxidation, which are quite difficult to be directly compared because of the different irradiation conditions and configurations, or because of the quite different thicknesses of either the WO_3_ and/or the BiVO_4_ photoactive layers in the heterojunctions.^29^ At the same time, the specific role played by nanostructuring on the overall PEC efficiency has never been disentangled by directly comparing the PEC performances attained with heterojunctions merely differing for their morphology.

Aiming at filling this gap, in this study we compare the water oxidation efficiency of WO_3_/BiVO_4_ heterojunction photoanodes displaying either a nanoflake-like or a planar morphology but similar film thickness and optical properties. Through a systematic investigation based on the use of several physicochemical and PEC characterization techniques, we demonstrate that the wavelength-dependent performance limitations of planar electrodes, observed with increasing thickness of the WO_3_ electron-carrier layer and leading to undesired photogenerated charge recombination,^20,30,31^ can be successfully overcome by structure control of the WO_3_ underlayer. Moreover, besides enhancing charge carriers separation on the 350–550 nm wavelength range, the here reported nanoflake-like heterojunction morphology allows preservation of the highly oxidizing valence band holes photoproduced in WO_3_ by ultraviolet (UV) light, with an overall 6-fold larger photogenerated current and charge separation efficiency.

## 2 Experimental Section

### 2.1 Chemicals and Materials

The following chemicals, all purchased from Sigma-Aldrich, were employed as supplied: tungstic acid (H_2_WO_4_, 99%), hydrogen peroxide (H_2_O_2_, 30 wt %), oxalic acid (HO_2_CCO_2_H, 99%), hydrochloric acid (HCl, 37 wt %), nitric acid (HNO_3_, 23.3 wt %), acetonitrile (CH_3_CN, 99%), bismuth(III) nitrate pentahydrate (Bi(NO_3_)_3_·5H_2_O, 98%), citric acid (99%), ammonium metavanadate (NH_4_VO_3_, 99%), acetylacetone (99%), ammonium metatungstate hydrate (99%, (NH_4_)_6_H_2_W_12_O_40_·*x*H_2_O), and poly(ethylene glycol) 300 (PEG 300). Poly(vinyl alcohol) (PVA) and urea (H_2_NCONH_2_, 99%) were purchased from Fluka. 2-Methoxyethanol (99%) was purchased from Alfa Aesar. 2 mm thick fluorine-doped tin oxide (FTO) glass (7 Ω/sq) was purchased from Kintec.

### 2.2 Photoelectrodes Preparation

#### 2.2.1 Planar Electrodes

A WO_3_ precursor solution was prepared by adding 0.494 g of ammonium metatungstate to 0.3 mL of acetylacetone and 0.7 mL of 2-methoxyethanol. The solution was heated at 50 °C and kept under stirring for 3 h; 50 mg of PEG 300 was finally added. A 2.5 × 2.5 cm^2^ FTO slab was coated with 100 μL of the so-obtained paste by spin-coating at 4000 rpm for 30 s. The final spinning rate was reached with a three-acceleration step program: 500 rpm s^–1^ up to 500 rpm, then 1500 rpm s^–1^ up to 2000 rpm, and finally 2000 rpm s^–1^ up to 4000 rpm. Prior to deposition, the FTO glass was cleaned by a 30 min long sonication in an aqueous soap solution and then in ethanol. After coating, the electrode was annealed at 550 °C for 1 h on a hot plate. The final annealing temperature was reached using a 1 h long heating ramp. This deposition step was repeated twice.

A BiVO_4_ precursor solution was prepared by adding 0.386 g of citric acid, 0.495 g of bismuth(III) nitrate, and 0.118 g of ammonium metavanadate to 3 mL of nitric acid. 100 μL of the paste was deposited at 4000 rpm for 30 s by spin-coating on a previously prepared WO_3_ photoelectrode, followed by annealing at 500 °C for 1 h, using the same hot plate and heating ramp as above. This deposition cycle was repeated 8 times. A reference photoanode containing only BiVO_4_ was prepared by performing the same deposition–calcination cyclic procedure 8 times on a clean FTO slab.

#### 2.2.2 Nanostructured Electrodes

WO_3_ nanostructured films on FTO were prepared according to the procedure described by Su et al.^32^ with some modifications. A ca. 200 nm thick seed layer was deposited on the FTO substrate by spin-coating 100 μL of a tungsten oxide precursor solution at 3000 rpm for 30 s. The solution was prepared by adding 0.417 g of tungstic acid and 0.167 g of PVA to 6 mL of 30% H_2_O_2_ followed by stirring for 3 h to obtain a transparent solution. The deposited film was then annealed for 2 h in a muffle furnace at 500 °C. The final annealing temperature was reached after a 1 h long heating ramp. WO_3_ nanoflakes (NF) were then grown on the seed layer by solvothermal synthesis. A 0.05 M tungstic acid precursor solution was prepared by adding 0.625 g of H_2_WO_4_ to 9 mL of 30% H_2_O_2_, and the volume was adjusted to 50 mL with distilled water. The so-obtained solution was kept at 80–85 °C for 30 min to allow the dissolution of the powders. The solution employed for the solvothermal synthesis was prepared as follows. 12 mL of the 0.05 M tungstic acid precursor solution, 0.08 g of oxalic acid, and 0.08 g of urea were dissolved in 50 mL of acetonitrile, followed by the addition of 1 mL of a HCl 37% aqueous solution. The solution was then placed in a 125 mL stainless steel autoclave, which was kept at 180 °C for 2 h. After the reaction, the electrodes were rinsed with water and calcined at 500 °C for 1 h in a muffle furnace, using a 1 h long heating ramp.

In order to obtain a nanostructured WO_3_/BiVO_4_ heterojunction, 60 μL of a BiVO_4_ solution was deposited at 4000 rpm for 30 s on the previously prepared WO_3__NF electrode, followed by annealing at 500 °C for 1 h in a muffle furnace. This BiVO_4_ deposition step was repeated twice in order to obtain a BiVO_4_ layer absorbing light similarly to the BiVO_4_ layer deposited on planar WO_3_ in the heterojunction.

The so-prepared photoanodes were labeled as WO_3__X, if consisting of WO_3_ only, and WO_3__X-BV, if consisting of a WO_3_/BiVO_4_ heterojunction, with X = P for planar WO_3_ underlayers or X = NF for nanoflake-like nanostructured WO_3_ underlayers. The photoanode containing BiVO_4_ only was labeled BV.

### 2.3 Optical, Morphological, Structural, and Photoelectrochemical Characterization

All optical absorption spectra were recorded by employing a Jasco V-670 spectrophotometer. The absorption spectra of optically transparent photoanodes with a planar surface (i.e., the planar WO_3_/BiVO_4_ heterojunction film and the BiVO_4_ reference film) were acquired in the transmittance mode. The absorption spectra of nanostructured electrodes were recorded in both the transmittance and reflectance modes, using an integrating sphere. Their absorbance *A* was then calculated from the reflectance *R* and the transmittance *T* values, using the following equation:^33^
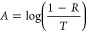
1The attainment of pure, crystalline materials was checked by X-ray diffraction (XRD) analysis, performed with a Rigaku Miniflex 600 diffractometer, equipped with a Cu tube providing Kα radiation. The diffractograms were background-corrected by employing the Origin software. A scanning electron microscope (SEM), model Jeol JSM-7600F with a 5 kV incident beam, was used to acquire top-view and cross-section images of planar and nanostructured WO_3_ and of the coupled WO_3_/BiVO_4_ electrodes.

Electrochemical impedance spectroscopy (EIS) data were acquired using an Autolab potentiostat controlled by Nova software. A 10 mV amplitude perturbation ranging from 10^5^ to 10^–1^ Hz was used. The light source was an LED-operating solar simulator (SUNBOX).^34^ The investigated electrodes, located in a 3-electrode quartz cell with a platinum wire counter electrode and a Ag/AgCl reference electrode, were irradiated in back configuration. Data were fit using ZView software.

Photoelectrochemical tests were carried out in a three-electrode cappuccino cell,^13^ with the photoanodes used as working electrodes (with an illuminated area of 0.28 cm^2^), Ag/AgCl (3 M KCl) as reference electrode, and a platinum gauze as counter electrode. The scan rate in linear sweep voltammetry (LSV) analyses was 10 mV s^–1^; the applied bias was controlled through an Autolab PGSTAT 12. The light source was a solar simulator (Newport, model Oriel LCS-100) equipped with an AM 1.5 G filter (light intensity fixed at 100 mW cm^–2^). All prepared electrodes were tested under both back-side irradiation, i.e., through the FTO glass, and front-side irradiation, i.e., through the photoactive material, in contact with a 0.5 M Na_2_SO_4_ electrolyte solution at pH 7. The potential vs Ag/AgCl values were converted into the RHE scale using the following equation: *E*_RHE_ = *E*_AgCl_ + 0.059 pH + *E*_AgCl_^°^, with *E*_AgCl_^°^ (3 M KCl) = 0.210 V at 25 °C.

Incident photon to current efficiency (IPCE) measurements were performed at 1.23 V vs RHE under monochromatic irradiation in the above-described three-electrode setup. The light source was a 300 W Lot-Oriel Xe lamp equipped with a Lot-Oriel Omni-λ 150 monochromator. Percent IPCE values were calculated using the following equation:
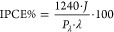
2where *J* is the recorded photocurrent density (mA cm^–2^) and *P*_λ_ (mW cm^–2^) is the power measured at each specific wavelength λ (nm). A 490 nm cutoff filter was used when recording photocurrents at λ > 500 nm, to cut any possible second harmonics contribution generated by the monochromator.

## 3 Results and Discussion

### 3.1 Morphological, Optical, and Structural Characterization of the Photoelectrodes

**Figure 1:**
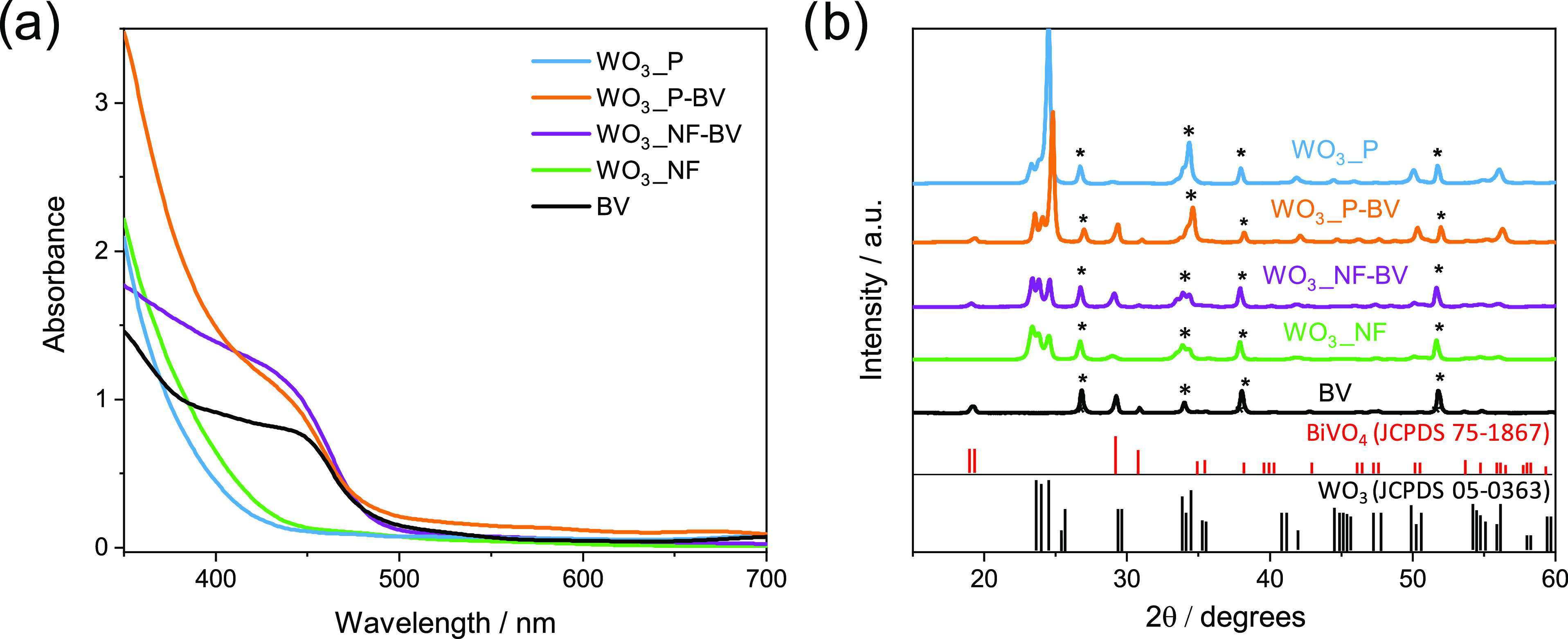
(a) Absorption spectra and (b) XRD patterns of planar and nanostructured WO_3_, WO_3_/BiVO_4_, and BiVO_4_ electrodes. The diffraction signals of WO_3_ (JCPDS 05–0363) and BiVO_4_ (JCPDS 75–1867) are reported for comparison; the peaks marked with an asterisk refer to FTO.

The optical absorption spectra of the prepared photoanodes are reported in Figure 1a. Planar and nanostructured WO_3_ electrodes exhibit a similar absorption profile, with an absorption onset at ca. 450 nm, accounting for a 2.7 eV band gap.^8^ The heterojunction electrodes exhibit higher absorption in the visible region, extending to ca. 500 nm, in excellent agreement with the 2.4 eV band gap of BiVO_4_.^35^ The absorption shoulders in the visible range, typical of BiVO_4_, are similar for the two heterojunction systems, indicating a comparable loading of bismuth vanadate on the underlying WO_3_ electrodes, independent of their morphology. The BV electrode exhibits a 500 nm absorption onset as the two heterojunction electrodes but a less intense shoulder at 450 nm, possibly due to a lower amount of BiVO_4_ directly deposited on FTO compared to that deposited on the planar or nanostructured WO_3_ underlayers.

The XRD patterns of the investigated systems are shown in Figure 1b. The two WO_3_ electrodes exhibit the typical peaks of a pure monoclinic structure,^36^ with the reflections at 23.4, 23.8, and 24.6° related to the {002}, {020}, and {200} planes, respectively, and the reflections at 33.5, 34, and 34.4° associated with the {022}, {−202}, and {202} planes.^14,37^ In the diffractogram of planar WO_3__P the intensity of the {200} and {202} peaks is higher compared to nanostructured WO_3__NF, suggesting a possible preferential orientation growth of tungsten oxide, or a more defective crystalline structure of WO_3__NF. WO_3__P also exhibits more intense reflections at 50.4 and 56.3°, associated with the {140} and {142} planes, respectively.^38^ A reference XRD pattern obtained with WO_3_ powders shows a relative intensity of the diffraction peaks more similar to those observed with WO_3__NF.^39^ The XRD patterns of the two heterojunctions show the coexistence of WO_3_ and BiVO_4_ reflections, this latter with crystalline monoclinic scheelite structure, as already reported,^11,40^ and evidenced by the 19.1, 29.1 and 31.1° reflections, corresponding to the {110}, {121}, and {040} planes, respectively.^33^

**Figure 2:**
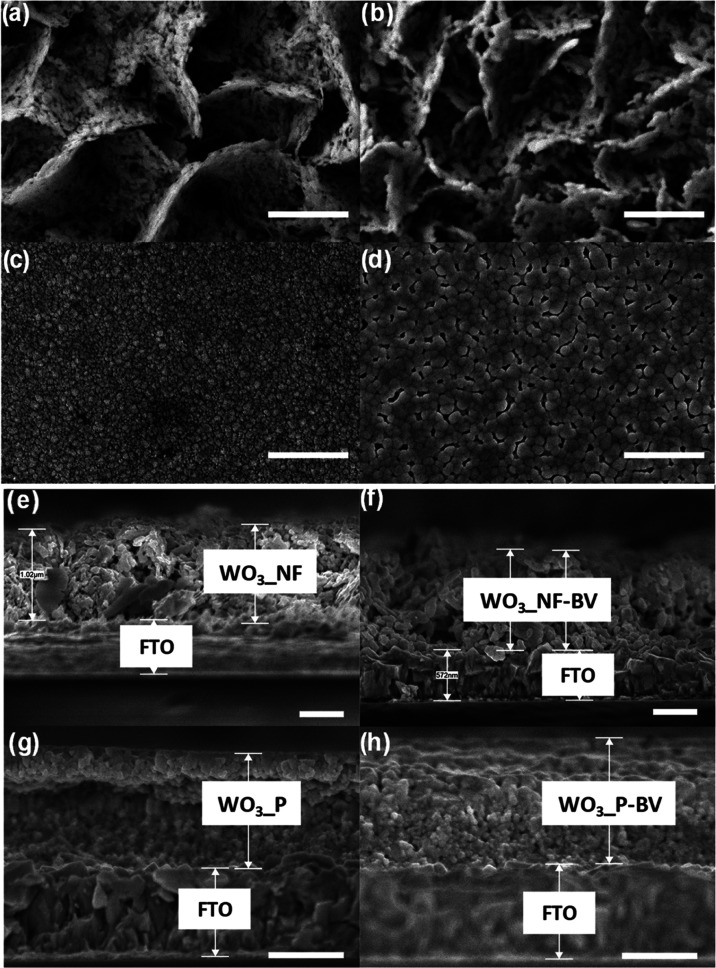
Top-view SEM images of (a) WO_3__NF, (b) WO_3__NF**-**BV, (c) WO_3__P, and (d) WO_3__P**-**BV electrodes; the scale bar is 1 μm. Cross-section SEM images of (e) WO_3__NF, (f) WO_3__NF**-**BV, (g) WO_3__P, and (h) WO_3__P**-**BV electrodes; the scale bar is 500 nm.

The top-view SEM images of the WO_3__NF films confirm the attainment of a nanostructured WO_3_ electrode (Figure 2a), characterized by three-dimensional (3D) structures with thin nanoflake thickness and abundant porosity. This morphology is maintained after BiVO_4_ deposition, as shown in Figure 2b. On the other hand, planar electrodes (Figure 2c,d) appear as a compact, aggregated network of particles, with no controlled morphology. In particular, the planar WO_3_ photoanode is mainly composed of relatively small and uniformly distributed spherical aggregates, while the top of the composite material, consisting of the BiVO_4_ layer, appears as a compact film characterized by a wormlike feature that completely covers the WO_3_ underlayer.^41^

The thicknesses of both nanostructured and planar electrodes, determined by averaging several cross-section values obtained from different SEM images (exemplified by Figure 2e–h), are reported in Table 1, together with the estimated standard deviation. Importantly, the thicknesses of planar WO_3_ and heterojunction electrodes are comparable to those of the corresponding nanostructured ones. Moreover, by subtracting the average film thickness of each WO_3_ electrode from that of the corresponding WO_3_/BiVO_4_ composite electrode, a similar thickness, ca. 130–150 nm, results for the BiVO_4_ layer deposited on both planar and nanostructured composite films, which is also confirmed by the comparable light absorption of the two heterojunctions in the visible range (Figure 1a).

**Table 1:** Thickness of the Overall WO_3_ and WO_3_/BiVO_4_ Photoactive Layers with Planar or NF Morphology, Estimated by Averaging Several Cross-Sectional Values Obtained from Different SEM Images

electrode	thickness (nm)
WO_3__NF	961 ± 62
WO_3__NF-BV	1126 ± 123
WO_3__P	790 ± 41
WO_3__P-BV	914 ± 18

**Scheme 1:**
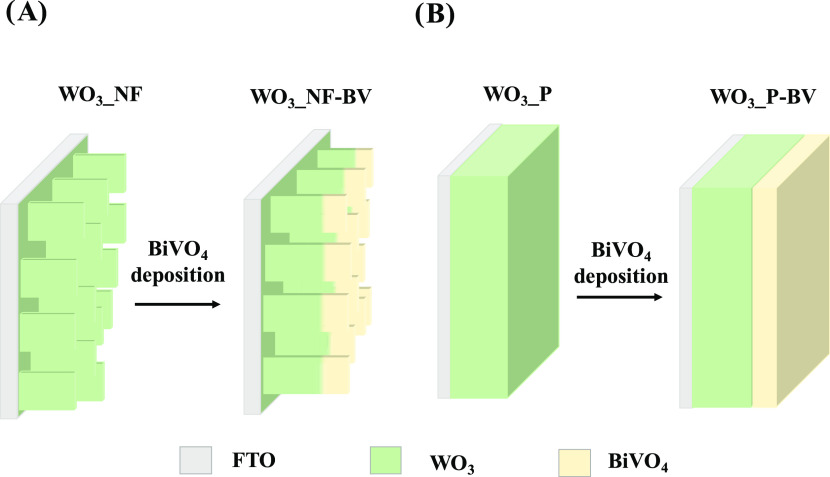
Schematic Illustration of (A) Nanostructured and (B) Planar WO_3_ and WO_3_/BiVO_4_ Photoanodes

A schematic illustration of the electrodes investigated here, resulting from their morphological characterization, is shown in Scheme 1.

### 3.2 PEC Characterization—Linear Sweep Voltammetry (LSV) Tests

**Figure 3:**
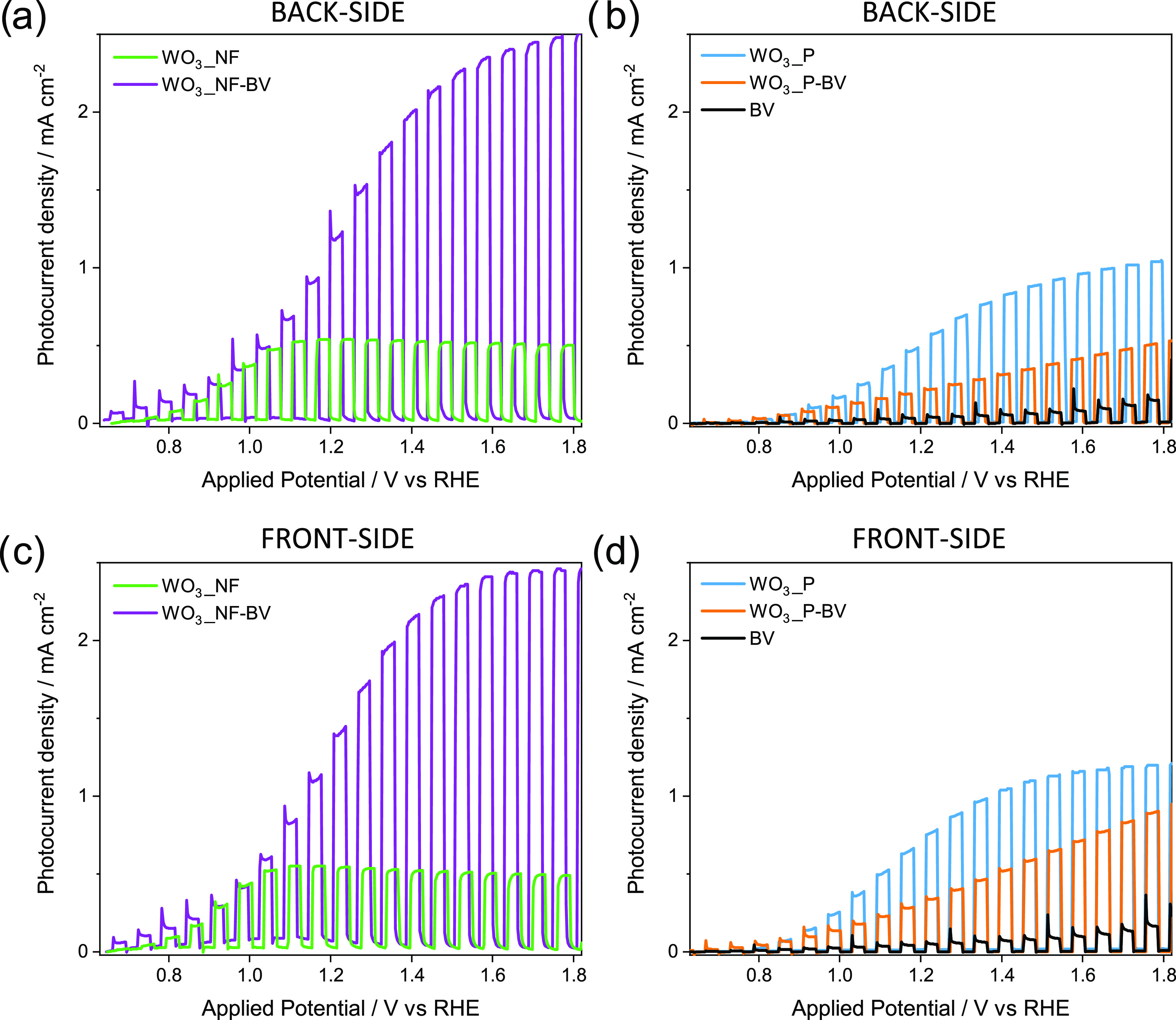
Linear sweep voltammetry (LSV) curves recorded with nanostructured and planar WO_3_, WO_3_/BiVO_4_, and BiVO_4_ electrodes, under (a, b) back-side and (c, d) front-side irradiation in 0.5 M Na_2_SO_4_.

The chopped linear sweep voltammetry (LSV) tests of pure and composite planar or nanoflake electrodes under back-side (i.e., across the FTO support) and front-side solar simulated irradiation can be compared in Figure 3. Five consecutive photocurrent density vs applied potential (*J*–*V*) scans were performed with each electrode. All of them provided a stable and reproducible photocurrent from the first to the last scan. Figure 3 reports the last acquired chopped LSV scans. The LSV plots recorded with the nanostructured electrodes (Figure 3a,3c) show that the saturation photocurrent generated by WO_3__NF is ca. 0.5 mA cm^–2^ under both front-side and back-side irradiation, in line with the results previously reported for electrodes with similar morphology and thickness.^36,42^ The slightly decreased photocurrent with applied voltage higher than 1.2 V_RHE_ can be related to the high density of electronic defects in this material, as suggested by XRD measurements (Figure 1b). These defects can lead to Fermi-level pinning, suppressing the beneficial effects of anodic bias on the recombination dynamics.^12^

Figure 3b,d report the LSV plots recorded with the planar WO_3_ and heterojunction electrodes. Notably, WO_3__P outperforms nanostructured WO_3__NF (see Figure 3a,b), reaching 1 mA cm^–2^ photocurrent under front-side irradiation at applied potentials larger than 1.2 V_RHE_. However, the photoactivity decreases upon deposition of BiVO_4_ on planar WO_3_, particularly under back-side irradiation (Figure 3b,d), and the photocurrent continuously increases with increasing applied potential. In contrast, the performance of WO_3__NF is considerably enhanced upon BiVO_4_ deposition. Indeed, as shown in Figure 3a,c, the WO_3__NF-BV heterojunction generates a photocurrent of ca. 1.3 and 2.5 mA cm^–2^ at 1.23 and 1.8 V_RHE_, respectively, with an up to 5-fold increase with respect to bare WO_3__NF. Moreover, comparable photocurrent values are attained under back-side (Figure 3a) and front-side (Figure 3c) irradiation.

The increased photocurrent can be explained by considering that BiVO_4_ extends the absorption onset of the photoactive system from 470 to 520 nm, allowing the exploitation of a wider spectral range of the incident light. Notably, at the same time, thanks to the favorable Fermi-level alignment between WO_3_ and BiVO_4_, the photocurrent onset of the composite WO_3__NF-BV electrode is sensibly anticipated to ∼0.6 V_RHE_,^43^ with respect to the 0.8 V_RHE_ onset observed for WO_3__NF. Moreover, in the case of WO_3__NF-BV, the photocurrent increases with the applied bias (Figure 3a,c) because the larger polarization favors charge carriers’ separation. However, the photocurrent generated by both heterojunction electrodes is higher compared to that of the reference BiVO_4_ electrode under both irradiation configurations, probably also due to the low thickness of the pure BiVO_4_ layer and the large recombination losses at the FTO/BiVO_4_ interface.^33^

**Scheme 2:**
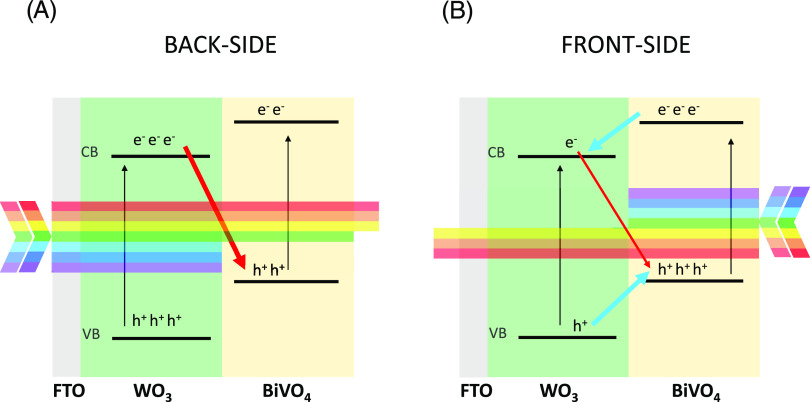
Schematic Illustration of the Irradiation Wavelength-Dependent Charge Recombination (Red Arrows) or Charge Separation (Blue Arrows) Paths at Work at WO_3_/BiVO_4_ Heterojunctions, under (A) Back-Side and (B) Front-Side Irradiation

Thus, LSV experiments (Figure 3) show that WO_3__NF-BV performs much better than WO_3__NF, and it is best performing in the electrode series under both back- and front-side irradiation. On the other hand, the deposition of a BiVO_4_ layer on planar WO_3__P leads to a lower photocurrent, particularly under back-side irradiation (Figure 3b,d). This effect was already highlighted by us and attributed to the undesired recombination between conduction band (CB) electrons in WO_3_ and valence band (VB) holes in BiVO_4_, at work in planar WO_3_/BiVO_4_ heterojunction photoanodes, especially when their WO_3_ layer is thicker than ca. 200 nm.^20,30,31^ This recombination path is indicated with red arrows in Scheme 2, which illustrates the prevailing charge transfer paths occurring in the WO_3_/BiVO_4_ heterojunction under front- and back-side irradiation.

Our LSV results can be fully understood in light of both the electrochemical impedance spectroscopy (EIS) tests under irradiation and the incident photon to current efficiency (IPCE) measurements discussed in the next sections.

### 3.3 Electrochemical Impedance Spectroscopy Tests

**Figure 4:**
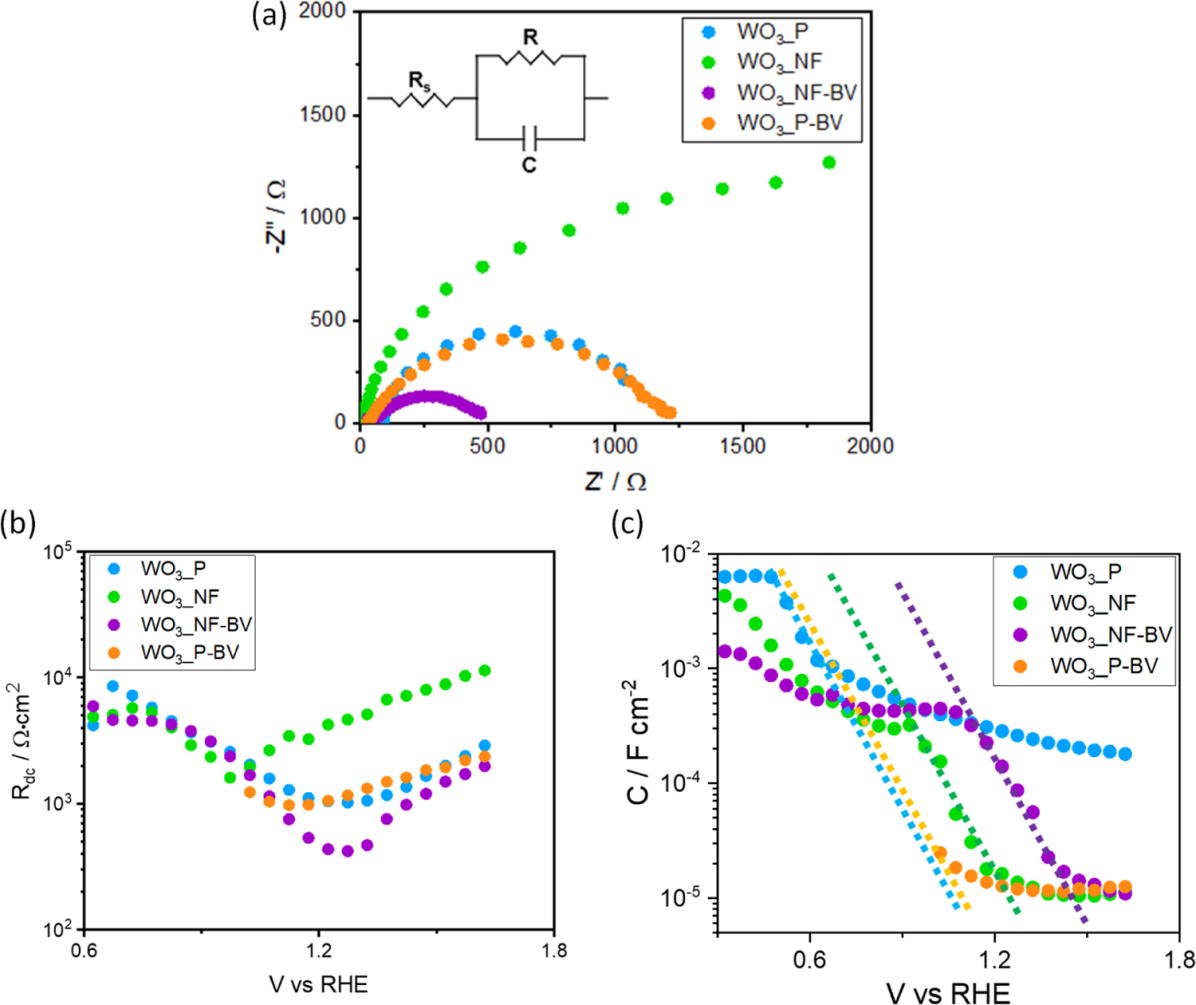
(a) Nyquist plots recorded with WO_3__P (blue), WO_3__NF (green), WO_3__NF-BV (purple), and WO_3__P-BV (orange) under 1 sun back-side irradiation, at 1.23 V_RHE_. Inset: Randles circuit. (b) Total resistance *R*_dc_ and (c) surface capacitance *C* of the same electrodes. The data obtained with WO_3__P–BV could be fitted only for applied potentials above 1 V_RHE_.

Figure 4a shows the Nyquist plots of all investigated samples under LED-based solar simulated back irradiation, at a single potential, 1.23 V_RHE_. The raw impedance data exhibit a single semicircle, which was fitted to a simple Randles’s equivalent circuit (Figure 4a, inset).^44,45^ In this circuit, *R*_s_ accounts for the series resistance of the electrochemical cell, including the contribution of the electrolyte, contacts, connection wires, etc., and is directly extracted from the high-frequency intercept (Z″= 0). On the other hand, the resistance *R* conveys the contribution of the charge transfer resistance at the semiconductor/liquid interface (*R*_ct_) and the transport resistance along the film (*R*_tr_), (*R* = *R*_ct_ + *R*_tr_) and is directly extracted from the diameter of the arc, although both individual resistances (*R*_ct_ and *R*_tr_) could not be deconvoluted from the impedance raw data. The total resistance (*R*_dc_) is obtained from the low-frequency intercept (Z″ = 0) (*R*_dc_ = *R*_s_ + *R*). On the other hand, *C* models the capacitance of the photoanode. This capacitance includes the double-layer capacitance and both the changes in the electron concentration in the conduction band and the variations in the electric field within the depletion region.

The two planar WO_3__P and WO_3__P-BV photoanodes display similar signals (Figure 4a), while the resistance of WO_3__P appears significantly lower compared to that of WO_3__NF at the water oxidation potential, indicating that the planar WO_3__P electrode drives more easily the overall water oxidation reaction. The highest resistance of nanostructured WO_3__NF is consistent with large surface recombination at the WO_3_/solution interface and may be responsible for the relatively low photocurrent values recorded under solar simulated irradiation (Figure 3a,c).

The evolution of both *R*_dc_ and *C* with applied potential for all tested samples are shown in Figure 4b,4c, respectively. Consistently with the behavior of the LSV curves, *R*_dc_ decreases with the applied potential down to a minimum value, coincident with the inflection point of the *J–V* curves and then increases at higher potentials. Although the data obtained with WO_3__P-BV could be fitted only for applied potentials above 1 V_RHE_, valuable information is still contained in the fitted data. The minimum *R*_dc_ value has been related to the extent of bulk recombination,^46^ and our results clearly show that, compared to WO_3__NF, bulk recombination in WO_3__NF-BV is minimized as a result of the enhanced charge separation upon addition of the BiVO_4_ layer. This clearly explains the significantly larger photocurrents observed for WO_3__NF-BV in Figure 3a,c. Conversely, no clear difference of the *R*_dc_ minimum is observed for the planar WO_3__P and WO_3__P-BV counterparts, indicating that a similar bulk recombination is present upon deposition of the BiVO_4_ layer on WO_3__P.

On the other hand, the capacitance of the photoanodes (Figure 4c) is mainly controlled by WO_3_ and shows a characteristic exponential evolution with applied potential, increasing at more cathodic potentials. This trend is typical of the chemical capacitance^47,48^ and reflects the exponential density of states near the conduction band.^46^ The dashed lines included in Figure 4c serve as an indication of the relative position of the WO_3_ conduction band for each studied photoanode. It is clear that for WO_3__NF the deposition of the BiVO_4_ layer leads to a positive shift of the semiconductor bands (reflected as the difference between the dashed green and purple lines in Figure 4c), which provides a higher thermodynamic driving force for WO_3_ holes to promote water oxidation. Conversely, this behavior is not observed in the planar WO_3__P photoanodes (no clear difference between the band positions after deposition of the BiVO_4_ layer), which can be due to Fermi-level pinning, evidencing the inefficient electron injection from BiVO_4_ into WO_3_, consistently with the decreased measured photocurrents of the planar heterostructure (Figure 3).

### 3.4 PEC Characterization—Incident Photon to Current Efficiency (IPCE) Tests

**Figure 5:**
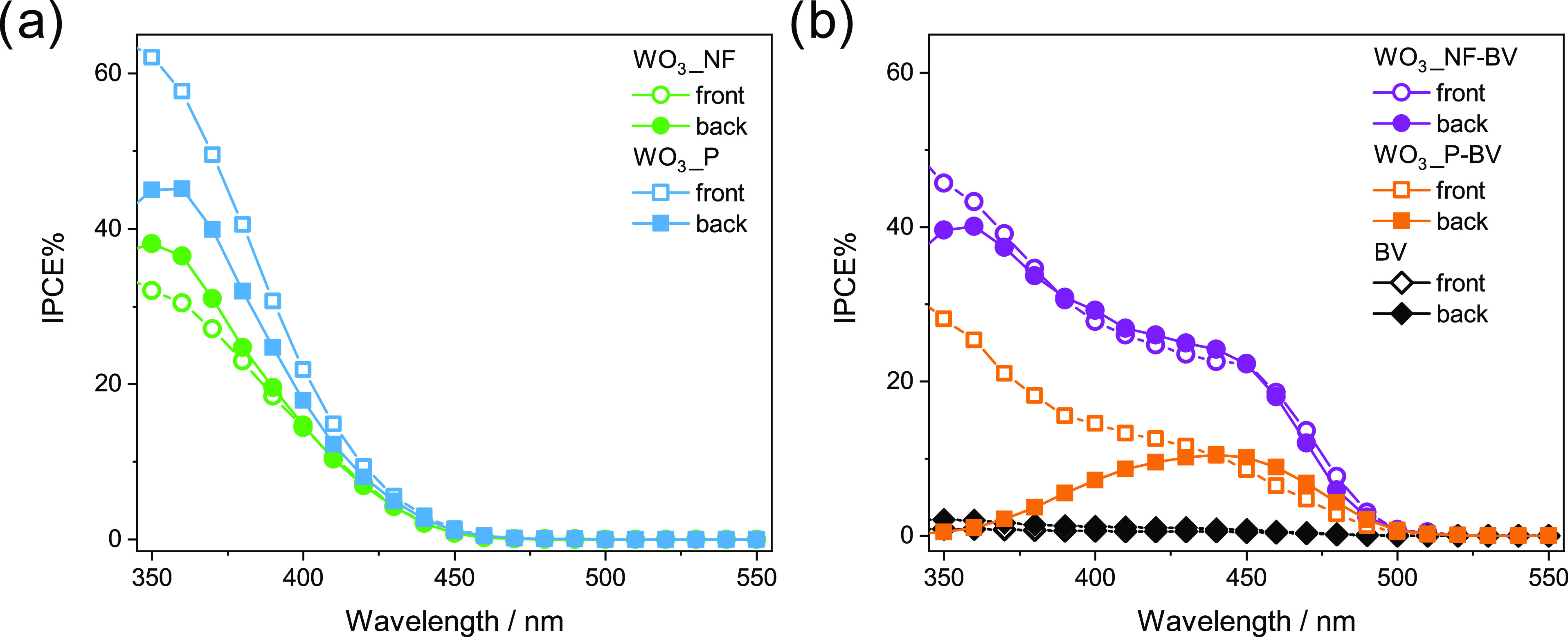
Incident photon to current efficiency (IPCE) curves obtained with (a) WO_3_ and (b) WO_3_/BiVO_4_ and BiVO_4_ electrodes at 1.23 V_RHE_ in Na_2_SO_4_ 0.5 M solution under back-side (full symbols) and front-side (open symbols) irradiation.

In order to determine the spectral signature of the photocurrent, IPCE measurements were recorded with the WO_3_, WO_3_/BiVO_4_ and BiVO_4_ electrodes at 1.23 V_RHE_ applied potential, under both back- and front-side monochromatic irradiation. The obtained IPCE curves are shown in Figure 5.

Both bare WO_3_ photoanodes exhibit a photocurrent onset at 450 nm (Figure 5a), consistent with the intrinsic band gap of WO_3_ (*E*_g_ = 2.7 eV).^30^ WO_3__P exhibits higher IPCE values compared to WO_3__NF and better performance under front-side irradiation (IPCE is 65% at 350 nm), in line with the results obtained under full lamp irradiation (see Figure 3b,d). The photoactivity loss under back-side irradiation is likely due to the poor hole mobility in WO_3_.^33^ In fact, in this configuration, the holes are photogenerated far from the electrolyte and largely recombine before reaching the electrode/electrolyte interface, whereas under front-side irradiation the holes are generated close to the extraction site. Differently, nanostructured WO_3__NF displays similar IPCE values in the two irradiation modes, implying an increased efficiency in hole consumption, in line with the average nanoflake width being smaller than the hole diffusion length in WO_3_ (∼150 nm).^49,50^ Indeed, the good performance of WO_3__NF under back-side illumination, when the holes are generated in the proximity of the FTO/WO_3_ interface, is related to the highly porous nanoflake-like structure, which allows an effective electrolyte penetration within the whole ca. 1 μm thick WO_3_ layer. The shorter distance between the hole generation sites and the hole collector electrolyte mitigates electron–hole pairs recombination.

The IPCE curves recorded with the composite materials (Figure 5b) evidence a photocurrent onset located at *ca*. 500 nm, in accordance with the visible light sensitization of WO_3_ achieved with BiVO_4_ (*E*_g_ = 2.4 eV) (see the absorption spectra in Figure 1a). While bare planar WO_3__P outperforms bare nanostructured WO_3__NF (Figure 5a), the WO_3__NF-BV heterojunction displays higher efficiency in the whole wavelength range and in both irradiation configurations. This is in line with the LSV results (Figure 3) and demonstrates that BiVO_4_ sensitization in the visible range is better exploited if the WO_3_ scaffold is nanostructured.

It is worth underlining that IPCE measurements were performed under a light intensity much lower than 1 sun. In order to compare the so-recorded photocurrent values with those determined in LSV analysis under AM 1.5 G irradiation conditions, we calculated the integrated photocurrent values from IPCE results by integrating the monochromatic efficiency over the global sunlight spectral irradiance. The so-calculated photocurrent *J* values are compared in Table 2 with the photocurrent values at 1.23 V_RHE_ measured in LSV plots (Figure 3).

**Table 2:** Comparison between the Photocurrent Values (*J*) at 1.23 V_RHE_ under Back- or Front-Side Irradiation, Calculated from IPCE Analyses or Obtained from LSV Analyses

	*J*@1.23 V_RHE_/mA cm^–2^ from LSV		*J*@1.23 V_RHE_/mA cm^–2^ from IPCE	
electrode	back	front	back	front
WO_3__NF	0.53	0.54	0.51	0.51
WO_3__P	0.57	0.77	0.62	0.86
WO_3__NF-BV	1.23	1.42	1.19	1.31
WO_3__P-BV	0.22	0.34	0.35	0.66

An outstanding agreement between the two sets of photocurrent values is obtained, for the whole set of investigated photoelectrodes, under both front- and back-side irradiation, the major difference being the slightly larger photocurrent values calculated for planar WO_3__P-BV under low irradiation intensity compared to the values measured in LSV analysis. This is in line with a larger charge recombination occurring in the planar system under high-intensity irradiation conditions.

The photocurrent values reported in Table 2 highlight that (i) photocurrents are always larger under front-side irradiation compared to back-side irradiation and that this effect is larger for planar WO_3__P and WO_3__P-BV electrodes; (ii) the photocurrents calculated from IPCE data are larger for planar electrodes and slightly smaller for the nanostructured ones, compared to LSV values; (iii) the deposition of BiVO_4_ on WO_3_ produces a photocurrent increase in the nanostructured electrode, which is slightly larger under front-side irradiation, and a photocurrent decrease in planar electrodes, particularly under back-side irradiation.

A detailed analysis of the IPCE curves reveals the different wavelength-dependent processes occurring in the two heterojunction architectures. The lower IPCE of the planar heterojunction under both irradiation conditions in the 350–450 nm range may be related to the more compact BiVO_4_ layer, which seems to act as a dielectric layer passivating the charge transfer from photoexcited WO_3_ to the solution.^30,51^

Furthermore, under back-side irradiation, the IPCE of planar WO_3__P-BV rapidly drops at short wavelengths (Figure 5b). Two effects concur with this efficiency loss. The first one occurs when both WO_3_ and BiVO_4_ absorb light, i.e., within the WO_3_ absorption edge (ca. 450 nm) and at wavelengths that filter through WO_3_ (e.g., 50% of 400 nm photons filter through planar WO_3_) and reach the BiVO_4_ layer.^26^ Under back-side irradiation at these wavelengths (400–450 nm), the electrons photopromoted in the conduction band of thick WO_3_ layers can recombine with the holes photogenerated in BiVO_4_ (see Scheme 2A), as demonstrated by the shortening of the BiVO_4_ holes lifetime evidenced by transient absorption spectroscopy.^20,52^

The second effect is due to the low hole diffusion in WO_3_ that prevails at short wavelengths, which are completely absorbed by the WO_3_ layer (WO_3_ absorbs more than 70% of incident photons below 380 nm, see Figure 1a). Consequently, the holes photogenerated in WO_3_ have limited access to the electrolyte because the thick and continuous BiVO_4_ overlayer further increases the distance between the photogeneration site and the hole extraction site. Thus, they accumulate at WO_3_ and more easily undergo recombination. For planar heterojunctions, lower performance is obtained compared to individual WO_3_ under both monochromatic and full lamp irradiation (Figures 3b,d and 5).

On the other hand, the WO_3__NF-BV electrode displays much higher IPCE compared to the planar heterojunction WO_3__P-BV electrode and negligible differences between the two irradiation modes (Figure 5b). Thus, the nanostructured architecture overcomes the efficiency losses burdening the planar structure. The maximum efficiency achieved with WO_3__NF-BV in the visible region (above 25% between 400 and 450 nm) is more than double compared to that of WO_3__P-BV. Furthermore, the enhanced IPCE attained in the 450–500 nm range indicates that the nanostructured architecture improves charge extraction from selectively photoexcited BiVO_4_.

At the same time, the very similar efficiency attained with WO_3__NF-BV at short wavelengths in the two irradiation modes demonstrates that the nanostructured WO_3_ scaffold hosts the BiVO_4_ coating, while retaining highly oxidant, accessible WO_3_ surface sites. This allows for better hole extraction from both semiconductors. In particular, the beneficial persistence of WO_3_ exposure to the electrolyte solution in the nanostructured heterojunction is further proved by the very similar IPCE values attained with bare WO_3__NF (Figure 5a) and combined WO_3__NF-BV (Figure 5b) electrodes at wavelengths shorter than 400 nm.

Thus, the peculiar morphology of this heterojunction electrode, besides preserving the oxidation photoactivity of the WO_3_ sublayer, prevents the recombination of the charge carriers photogenerated when both semiconductors are excited in the 400–450 nm wavelength range, by favoring electron extraction to the external circuit through WO_3_ and hole consumption at the electrode/electrolyte interface.

The excellent activity of WO_3__NF-BV can be better highlighted by comparing the IPCE values of each WO_3_/BiVO_4_ heterojunction electrode recorded under back-side irradiation with those obtained with the corresponding single components, by calculating the so-called IPCE enhancement factor^30^
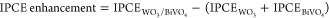
3where IPCE_WO_3_/BiVO_4__ is the IPCE measured with the coupled systems and IPCE_WO_3__ and IPCE_BiVO_4__ are the IPCEs of the corresponding bare WO_3_ and of the ca. 130 nm thick pure BiVO_4_ electrode, respectively, recorded in separate experiments.

**Figure 6:**
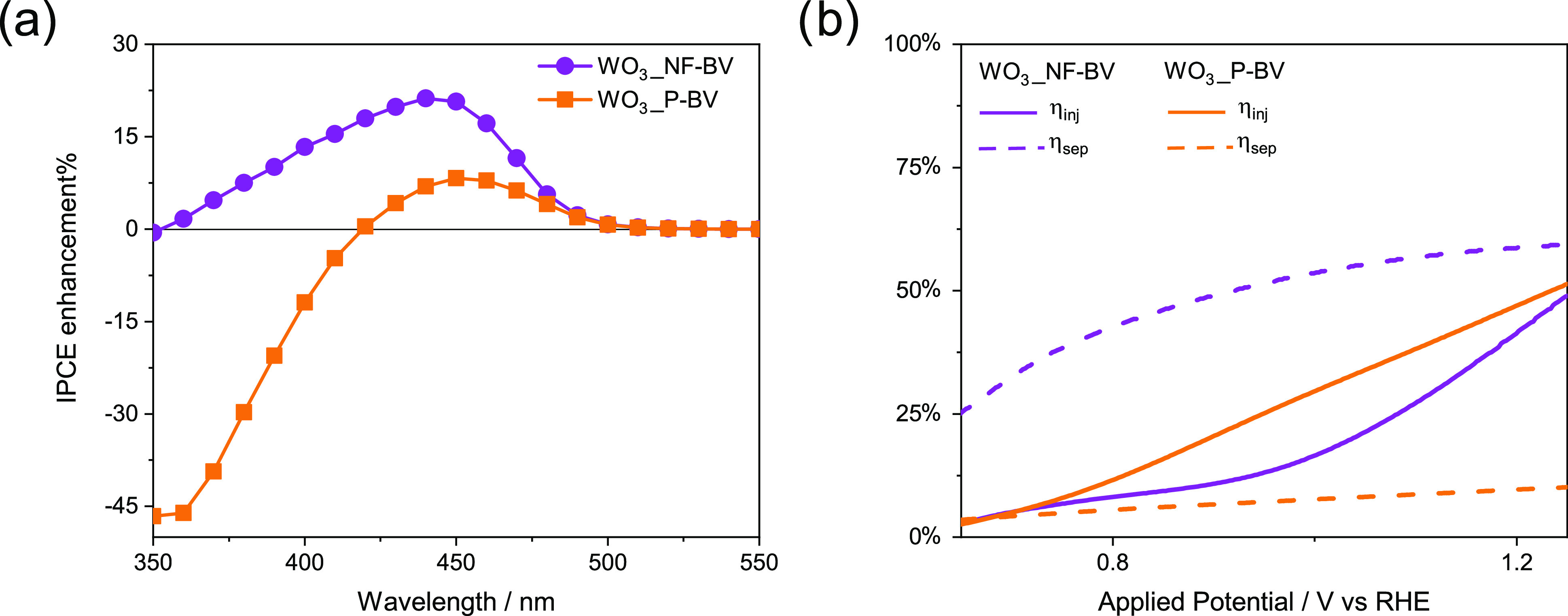
(a) IPCE enhancement factor calculated according to eq 3. (b) Charge injection (continuous lines) and charge separation efficiency (dashed lines) of the nanostructured (purple) and planar (orange) WO_3_/BiVO_4_ heterojunctions.

Figure 6a shows that the planar WO_3__P-BV electrode does not maintain the performance of its underlying WO_3_ layer in the UV region. Thus, its IPCE enhancement factor is negative at wavelengths shorter than 420 nm, attaining a ca. −45% value at 350 nm (orange curve in Figure 6a). Moreover, the IPCE enhancement in the visible light region is quite modest, reaching a maximum of 7% at 450 nm. Differently, the nanostructured WO_3__NF-BV heterojunction generates an enhancement factor in the 350–500 nm range, with a 25% maximum at 450 nm and a progressive reduction with decreasing irradiation wavelength, becoming zero at 350 nm.

### 3.5 Charge Separation and Charge Injection Efficiencies

Simulated solar light and monochromatic PEC experiments point toward the role played by the WO_3_ nanostructured underlayer in increasing the bulk charge separation efficiency or the surface charge injection efficiency in the heterojunction electrode. To further investigate these aspects, we performed experiments under back-side irradiation in the presence of sulfite anions, acting as sacrificial oxidation agents, to calculate the photogenerated charge separation efficiency (η_sep_) in the bulk and the minority charge carrier injection efficiency at the electrode/electrolyte interface (η_inj_) of the electrodes.

In fact, the water oxidation photocurrent density can be expressed as *J* = *J*_abs_·η_sep_·η_inj_, where *J*_abs_ is the maximum theoretical current density, calculated from the integration of the product between the standard AM 1.5 G solar spectrum and the absorption spectrum of the electrode, over the proper absorption wavelength range of the film.^53^ By assuming a 100% charge injection efficiency for Na_2_SO_3_ oxidation (η_inj_ = 1), the photocurrent attained with our electrodes in a 0.5 M Na_2_SO_3_ electrolyte buffered at pH 7 (*J*_Na_2_SO_3__) is *J*_Na_2_SO_3__ = *J*_abs_·η_sep_, from which η_sep_ can be simply obtained by dividing the photocurrent measured in the presence of the Na_2_SO_3_ hole scavenger by *J*_abs_. From the combination of the two photocurrent expressions (in the presence and absence of the hole scavenger), one can finally calculate η_inj_, which is equal to the ratio between *J*_H_2_O_ and *J*_Na_2_SO_3__.

The calculated η_sep_ and η_inj_ values for the two heterojunction electrodes are reported in Figure 6b as a function of the applied potential. The charge injection efficiency η_inj_ of both heterojunctions progressively increases with the applied potential up to the water oxidation potential, when η_inj_ is ∼50% for both heterojunctions. This behavior is clearly ascribed to the BiVO_4_ top layer in both heterojunctions and to its low efficiency in charge transfer to the solution.^54^

On the other hand, the nanostructured WO_3__NF-BV heterojunction electrode clearly exhibits a 6-fold higher charge separation efficiency compared to the planar WO_3__P-BV heterojunction (Figure 6b), with η_sep_ values of ∼10 and ∼60% at 1.23 V_RHE_ for WO_3__P-BV and WO_3__NF-BV, respectively, which is consistent with the resistance values shown in Figure 4b and with the pinning of the bands shown by the capacitance (Figure 4c). Thus, the performance of the WO_3__NF-BV electrode is much less limited by charge recombination in the bulk, in line with EIS results, which confirms the key role that morphology tuning has in the photogenerated charge transport properties of these materials.

## 4 Conclusions

WO_3_ nanostructuring plays a pivotal role in enhancing the overall performance attained with WO_3_/BiVO_4_ heterojunctions by overcoming the undesired limitations of planar composites. Two main factors lead to the substantial increase of PEC activity of nanostructured with respect to planar systems: (i) an improved charge separation efficiency due to the nanoflake-like arrangement of the underlying WO_3_ layer that allows a better electron extraction toward the external circuit, while the holes photogenerated at the semiconductors interface can be easily transferred to the electrolyte, and (ii) the preservation of exposed tungsten trioxide surface in direct contact with the electrolyte also after the BiVO_4_ layer deposition. The accessibility to the electrolyte throughout the whole thickness of the photoactive layer, provided by the open structure and the good electron transport property of the composite nanostructured system, ensures efficient electron/hole separation and hole consumption under both front-side and back-side irradiation and in the whole wavelengths range. WO_3_ nanostructuring is a promising strategy to fully exploit the beneficial effects of the WO_3_/BiVO_4_ type-II heterojunction and to suppress detrimental recombination paths of photogenerated charge carriers, which instead may be active in planar WO_3_/BiVO_4_ systems.
